# Supplementary material for: PROmotion of COvid-19 VA(X)ccination in the Emergency Department—PROCOVAXED: study protocol for a cluster randomized controlled trial
Source: Trials. 2022 Apr 21;23:332. doi: 10.1186/s13063-022-06285-x (PMC9021557; doi:10.1186/s13063-022-06285-x)
Supplement: Supplementary file 5 — Additional file 5. UCSF Verbal consent – Intervention Group. [file 13063_2022_6285_MOESM5_ESM.pdf]

UNIVERSITY OF CALIFORNIA, SAN FRANCISCO INFORMATION FOR PARTICIPATION IN A RESEARCH STUDY

Study Title: PROmotion of COvid-19 VA(X)ccination in the Emergency Department – PROCOVAXED

Why is this study being done?

This is a survey study. The study doctor(s), Dr. Rodriguez, the research coordinator, or research assistants, will explain this study to you.

The purpose of this study is to find out whether providing you information about Covid vaccines in the Emergency Department increases acceptance of these vaccines.

Taking part in this survey is your choice. No matter what decision you make, there will be no penalty to you and your decision will not affect your medical care. Take your time to make your decision about participating. This study is funded by The National Institutes of Health.

How many people will take part in this study?

About 1,260 people will take part in this survey.

What will happen if I take part in this research study?

We will ask you several survey questions now and several survey questions at the end of your visit here in the emergency department. This will not affect your evaluation and treatment in the emergency department. The survey is considered a research procedure and consists of questions regarding the COVID vaccine and the information you received about the vaccine in the ED today.

How long will I be in the study?

This study will take a total of about 12 minutes to complete (7 minutes for the first survey and 5 minutes for the second) and will not interfere with or prolong your time in the Emergency Department.

Can I stop being in the survey?

Yes, you can stop at any time. You do not have to answer any questions that you do not want to. You can do one survey and not the other if you want.

What side effects or risks can I expect from being in the study?

- The risks associated with this study are minimal, but include a potential loss of privacy.

- We will do our best to make sure that the personal information gathered for this study is kept private. However, we cannot guarantee total privacy. Your personal information may be given out if required by law. If information from this study is published or presented at scientific meetings, your name and other personal information will not be used. You are free to skip any question.

Are there benefits to taking part in the study?

There will be no direct benefit to you from participating in this study. However, the information that you provide may help health professionals learn more about how to address Covid-19 vaccine hesitancy.

Will information about me be kept private?

We will do our best to make sure that the personal information gathered for this study is kept private. However, we cannot guarantee total privacy. Your personal information may be given out if required by law. If information from this study is published or presented at scientific meetings, your name and other personal information will not be used. Organizations that may look at/copy your research records for research, quality assurance, and data analysis include the Representatives of the University of California and The National Institute of Health.

This research is covered by a Certificate of Confidentiality from the National Institutes of Health. This means that the researchers cannot release or use information, documents, or samples that may identify you in any action or suit unless you say it is okay. They also cannot provide them as evidence unless you have agreed. This protection includes federal, state, or local civil, criminal, administrative, legislative, or other proceedings. An example would be a court subpoena.

There are some important things that you need to know. The Certificate DOES NOT stop reporting that federal, state or local laws require. Some examples are laws that require reporting of child or elder abuse, some communicable diseases, and threats to harm yourself or others. The Certificate CANNOT BE USED to stop a sponsoring United States federal or state government agency from checking records or evaluating programs. The Certificate DOES NOT stop disclosures required by the federal Food and Drug Administration (FDA). The Certificate also DOES NOT prevent your information from being used for other research if allowed by federal regulations.

Researchers may release information about you when you say it is okay. For example, you may give them permission to release information to insurers, medical providers or any other persons not connected with the research. The Certificate of Confidentiality does not stop you from willingly releasing information about your involvement in this research. It also does not prevent you from having access to your own information.

How will my information be used?

Researchers will use your information to conduct this study. Once the study is done using your information, we may share it with other researchers so they can use it for other studies in the future. We will not share your name or any other personal information that would let the researchers know who you are. We will not ask you for additional permission to share this deidentified information.

What are the costs of taking part in this study?

There are no charges to you from this study.

Will I be paid for taking part in this study?

You will not be paid for taking part in this study.

Who can answer my questions about the study?

You can talk to the researcher(s) about any questions, concerns, or complaints you have about this study. Contact the researcher: Dr. Robert M. Rodriguez MD at 628-206-5875

If you wish to ask questions about the study or your rights as a research participant to someone other than the researchers or if you wish to voice any problems or concerns you may have about the study, please call the Institutional Review Board at 415-476-1814.
