# Supplementary material for: PROmotion of COvid-19 VA(X)ccination in the Emergency Department—PROCOVAXED: study protocol for a cluster randomized controlled trial
Source: Trials. 2022 Apr 21;23:332. doi: 10.1186/s13063-022-06285-x (PMC9021557; doi:10.1186/s13063-022-06285-x)
Supplement: Supplementary file 6 — Additional file 6. UCSF Follow-Up Phone Call. [file 13063_2022_6285_MOESM6_ESM.pdf]

UNIVERSITY OF CALIFORNIA, SAN FRANCISCO
CONSENT TO PARTICIPATE IN A RESEARCH STUDY

Study Title: PROmotion of COvid-19 VA(X)ccination in the Emergency Department – PROCOVAXED

Research Project Director:	Robert Rodriguez, M.D., Professor of Emergency Medicine UCSF, Room 809, 505 Parnassus Ave, San Francisco, CA. Phone: 628 206-5875 e-mail: Robert.rodriguez@ucsf.edu

Study Coordinator:	Virginia Chan, virginia.chan@ucsf.edu

This is a research study about *COVID-19 and Vaccines*. The study researchers, **Dr. Robert Rodriguez, MD** from the UCSF Department of Emergency Medicine *w*ill explain this study to you.

DETAILED STUDY INFORMATION

This part of the consent form gives you more detailed information about what the study involves.

Research studies include only people who choose to take part. Please take your time to make your decision about participating and discuss your decision with your family or friends if you wish. If you have any questions, you may ask the researchers.

You are being asked to take part in this study because you have received care at the UCSF Parnassus or The Zuckerberg San Francisco General Emergency Department.

Why is this study being done?

This is a follow up study. The study doctor(s), Dr. Rodriguez, the research coordinator, or research assistants, will explain this study to you.

The purpose of this study is to find out whether providing you information about Covid vaccines in the Emergency Department increases acceptance of these vaccines.

Taking part in this follow up study is your choice. No matter what decision you make, there will be no penalty to you and your decision will not affect your medical care. Take your time to make your decision about participating.

How many people will take part in this study?

About1,260 people will take part.

What will happen if I take part in this research study?

We will take down your phone number and call you in about a month to ask if you have received the Covid vaccine. We will also review your medical record one month past your ED visit to see if you have received a Covid vaccine. This will not affect your evaluation and treatment in the emergency department or your other healthcare anywhere.

How long will I be in the study?

The phone call follow up will take a total of about 5 minutes to complete. It will not affect any of your future healthcare. We will not contact you after these calls without your separate approval.

Can I stop being in the study?

Yes. You can decide to stop at any time. Just tell the study researcher or staff person right away if you wish to stop being in the study. You do not have to answer any of the questions you do not want to answer.

Also, the study researcher may stop you from taking part in this study at any time if he or she believes it is in your best interest, if you do not follow the study rules, or if the study is stopped.

What side effects or risks can I expect from being in the study?

- The risks associated with this study are minimal, but include a potential loss of privacy.
- We will do our best to make sure that the personal information gathered for this study is kept private. However, we cannot guarantee total privacy. Your personal information may be given out if required by law. If information from this study is published or presented at scientific meetings, your name and other personal information will not be used. You are free to skip any question.

Are there benefits to taking part in the study?

There will be no direct benefit to you from participating in this study. However, the information that you provide may help health professionals learn more about how to address Covid-19 vaccine hesitancy.

What other choices do I have if I do not take part in this study?

You are free to choose not to participate in the study. If you decide not to take part in this study, there will be no penalty to you. You will not lose any of your regular benefits, and you can still get your care from our institution the way you usually do.

How will my information be used?

Researchers will use your information to conduct this study. Information gathered during this research study will only be used for this study. They will not be shared with other researchers.

Will information about me be kept private?

We will do our best to make sure that the personal information gathered for this study is kept private. However, we cannot guarantee total privacy. Your personal information may be given out if required by law. If information from this study is published or presented at scientific meetings, your name and other personal information will not be used.

Authorized representatives from the following organizations may review your research data for the purpose of monitoring or managing the conduct of this study:

- Representatives of the National Institutes of Health
- Representatives of the University of California

This research is covered by a Certificate of Confidentiality from the National Institutes of Health. This means that the researchers cannot release or use information, documents, or samples that may identify you in any action or suit unless you say it is okay. They also cannot provide them as evidence unless you have agreed. This protection includes federal, state, or local civil, criminal, administrative, legislative, or other proceedings. An example would be a court subpoena.

There are some important things that you need to know. The Certificate DOES NOT stop reporting that federal, state or local laws require. Some examples are laws that require reporting of child or elder abuse, some communicable diseases, and threats to harm yourself or others. The Certificate CANNOT BE USED to stop a sponsoring United States federal or state government agency from checking records or evaluating programs. The Certificate DOES NOT stop disclosures required by the federal Food and Drug Administration (FDA). The Certificate also DOES NOT prevent your information from being used for other research if allowed by federal regulations.

Researchers may release information about you when you say it is okay. For example, you may give them permission to release information to insurers, medical providers or any other persons not connected with the research. The Certificate of Confidentiality does not stop you from willingly releasing information about your involvement in this research. It also does not prevent you from having access to your own information.

Are there any costs to me for taking part in this study?
There are no charges to you from this study.

Will I be paid for taking part in this study?
You will not be paid for taking part in this study.

What are my rights if I take part in this study?

Taking part in this study is your choice. You may choose either to take part or not to take part in the study. If you decide to take part in this study, you may leave the study at any time. No

matter what decision you make, there will be no penalty to you in any way. You will not lose any of your regular benefits, and you can still get your care from our institution the way you usually do.

Who can answer my questions about the study?

You can talk to the researcher(s) about any questions, concerns, or complaints you have about this study. Contact the researcher Dr. Robert Rodriguez at 628-206-5875

If you wish to ask questions about the study or your rights as a research participant to someone other than the researchers or if you wish to voice any problems or concerns you may have about the study, please call the Institutional Review Board at 415-476-1814

CONSENT

You have been given a copy of this consent form to keep. You will be asked to sign a separate form authorizing access, use, creation, or disclosure of health information about you.

PARTICIPATION IN RESEARCH IS VOLUNTARY. You have the right to decline to be in this study, or to withdraw from it at any point without penalty or loss of benefits to which you are otherwise entitled.

If you wish to participate in this study, you should sign below.

____________ ____
Date Participant's Signature for Consent

____________ ____
Date Person Obtaining Consent
