# Supplementary material for: PROmotion of COvid-19 VA(X)ccination in the Emergency Department—PROCOVAXED: study protocol for a cluster randomized controlled trial
Source: Trials. 2022 Apr 21;23:332. doi: 10.1186/s13063-022-06285-x (PMC9021557; doi:10.1186/s13063-022-06285-x)
Supplement: Supplementary file 7 — Additional file 7. PROCOVAXED HIPAA Form. [file 13063_2022_6285_MOESM7_ESM.pdf]

IRB# 21-34004

University of California, San Francisco (UCSF Health)
Permission to Use Personal Health Information for Research

Study Title (or IRB Approval Number if study title may breach Participant's privacy):
PROmotion of COvid-19 VA(X)ccination in the Emergency Department – PROCOVAXED

Principal Investigator Name: Robert Rodriguez, MD

Sponsor/Funding Agency (if funded): NIH

A. What is the purpose of this form?

State and federal privacy laws protect the use and release of your health information. Under these laws, the University of California or your health care provider cannot release your health information for research purposes unless you give your permission. Your information will be released to the research team which includes the researchers, people hired by the University or the sponsor to do the research and people with authority to oversee the research. If you decide to give your permission and to participate in the study, you must sign this form as well as the Consent Form. This form describes the different ways that UCSF Health can share your information with the researcher, research team, sponsor and people with oversight responsibility. The research team will use and protect your information as described in the attached Consent Form. However, once your health information is released by UCSF Health it may not be protected by the privacy laws and might be shared with others. If you have questions, ask a member of the research team.

B. What Personal Health Information will be released?

If you give your permission and sign this form, you are allowing UCSF Health to release the following medical records containing your Personal Health Information. Your Personal Health Information includes health information in your medical records, financial records and other information that can identify you.

☐ Entire Medical Record	☐ Lab & Pathology Reports	■ Emergency Dept. Records
☐ Ambulatory Clinic	☐ Dental Records	☐ Financial records
☐ Progress Notes	☐ Operative Reports	☐ Imaging Reports
■ Other Test Reports	☐ Discharge Summary	☐ History & Physical Exams
■ Other (describe):	☐ Consultation	☐ Psychological Tests

COVID Test results

C. Do I have to give my permission for certain specific uses?

Yes.

☐ The research team will also be collecting information from your medical record that is marked by the check box. The following information will only be released if you give your specific permission by putting your initials on the line(s).

- ☐ I agree to the release of information pertaining to drug and alcohol abuse, diagnosis or treatment. ________(initials)
- ☐ I agree to the release of HIV/AIDS testing information.________ (initials)
- ☐ I agree to the release of genetic testing information._______ (initials)
- ☐ I agree to the release of information pertaining to mental health diagnosis or treatment.______ (initials)

D. Who will disclose and/or receive my Personal Health Information?

Your Personal Health Information may be shared with these people for the following purposes:

1. To the research team for the research described in the attached Consent Form;
2. To others at UC with authority to oversee the research
3. To others who are required by law to review the quality and safety of the research, including: U.S. government agencies, such as the Food and Drug Administration or the Office of Human Research Protections, the research sponsor or the sponsor's representatives including but not limited to the contract research organization (CRO), or government agencies in other countries.

E. How will my Personal Health Information be shared for the research?

If you agree to be in this study, the research team may share your Personal Health Information in the following ways:

1. To perform the research
2. Share it with researchers in the U.S. or other countries;
3. Use it to improve the design of future studies;
4. Share it with business partners of the sponsor; or
5. File applications with U.S. or foreign government agencies to get approval for new drugs or health care products.

F. Am I required to sign this document?

No, you are not required to sign this document. You will receive the same clinical care if you do not sign this document. However, if you do not sign the document, you will not be able to participate in this research study.

G. Optional research activity

■ There are no optional research activities.

☐ The research I am agreeing to participate in has additional optional research activity such as the creation of a database, a tissue repository or other activities, as explained to me in the informed consent process, I understand I can choose to agree to have my information shared for those activities or not.

I agree to allow my information to be disclosed for the additional optional research activities explained in the informed consent process.______________(initials)

H. Does my permission expire?

This permission to release your Personal Health Information expires when the research ends and all required study monitoring is over.

I. Can I cancel my permission?

You can cancel your permission at any time. You can do this in two ways. You can write to the researcher or you can ask someone on the research team to give you a form to fill out to cancel your permission. If you cancel your permission, you may no longer be in the research study. You may want to ask someone on the research team if canceling will affect your medical treatment. If you cancel, information that was already collected and disclosed about you may continue to be used for limited purposes. Also, if the law requires it, the sponsor and government agencies may continue to look at your medical records to review the quality or safety of the study.

J. Signature

Participant

If you agree to the use and release of your Personal Health Information, please print your name and sign below. You will be given a signed copy of this form.

____
Participant's Name (print)--*required*

____ ____________________
Participant's Signature Date

Parent or Legally Authorized Representative

If you agree to the use and release of the above named Participant's Personal Health Information, please print your name and sign below.

____

Parent or Legally Authorized Representative's Name (print) Relationship to the Participant

____

Parent or Legally Authorized Representative's Signature Date

Witness

If this form is being read to the Participant because s/he cannot read the form, a witness must be present and is required to print his/her name and sign here:

____

Witness' Name (print)

______________________________________ ____________________

Witness' Signature Date

Instructions for Researchers: This version clarifies Instructions for Researchers Item 3b.There are no other changes to the document. Do not make any changes to this form except where fillable fields allow entries.

The IRB will not be confirming the accuracy of the information you complete on this form. The researchers are responsible for accurately completing the HIPAA Research Authorization as follows:

- Page 1, Item B: Mark all sources of PHI that will be released
- Page 2, Item C:
  a. Check the first box if any of the 4 categories of sensitive information will be collected
  b. Then, check the box only for each specific type of information that will be collected for this study
  c. Obtain the participant's initials only for the specific types of information
- Page 3, Item G:
  a. Check one of the boxes indicating if there are optional research activities or not
  b. Obtain the participant's initial only if the study involves optional research activity, and the participant agrees to the optional research activity.
- Page 3, Item J: Obtain the participant's name, signature, and date; complete subsequent signature lines if applicable
- Provide the Participant with a signed copy of the form

  Note: Do not include the last page (Page 5) when distributing this document to study participants. Save, print, and distribute pages 1-4. To save time, pre-fill and save this form (except for the study title and IRB #).
